# The Burden of MDR/XDR Tuberculosis in Coastal Plains Population of China

**DOI:** 10.1371/journal.pone.0117361

**Published:** 2015-02-17

**Authors:** Xiujun Yang, Yanli Yuan, Yu Pang, Bo Wang, Yunlong Bai, Yanhua Wang, Baozhu Yu, Zhiying Zhang, Ming Fan, Yanlin Zhao

**Affiliations:** 1 Jilin Province Center for Disease Control and Prevention, Changchun, China; 2 Chinese Center for Disease Control and Prevention, Beijing, China; 3 PATH Office, Beijing, China; Hospital San Agustín. Aviles. Asturias. Spain, SPAIN

## Abstract

**Background:**

We conducted a first baseline survey in Jilin Province of China to determine the proportion of drug-resistant tuberculosis (TB), and to analyze risk factors associated with the emergence of drug-resistance.

**Methodology/Principal Findings:**

Thirty counties in Jilin Province were randomly selected as survey sites using a stratified cluster sampling method. People enrolled in the survey were new and re-treated, smear-positive pulmonary TB patients newly enrolled in local TB control and prevention institutions during the survey period. Sputum samples were collected, and the susceptibility of bacterial strains to anti-TB drugs was analyzed by proportion method. Based on the survey results, we estimated the number of drug-resistant TB patients and analyzed the risk factors associated with the emergence of drug resistance. Of 1,174 new TB patients and 597 re-treated TB patients, 8.6% and 23.2% were multi-drug resistant (MDR)-TB patients, respectively. Approximately 12% of MDR-TB patients were extensively drug-resistant. We estimate that approximately 1,290 new MDR-TB cases develop in Jilin Province every year. Of these, 810 cases would be new patients, and 480 cases would involve re-treated patients. Risk factors associated with MDR-TB include employment status, educational background, and income level.

**Conclusions/Significance:**

Jilin Province remains one of the highest-burden areas in China for drug-resistant TB. The higher number of MDR-TB among new cases suggested that the transmission of drug-resistant strains in Jilin is an urgent problem in the MDR-TB control and prevention system of Jilin Province. Improving the treatment compliance of TB patients and the quality of medical care in public health institutions is urgently needed.

## Introduction

China has the second-highest burden of tuberculosis (TB) in the world [1–4]. According to WHO estimates, approximately 1 million new TB cases and 54,200 MDR-TB cases in 2013 [5] occur in China. In 2007–08, China conducted a nationwide drug-resistance survey, and the results showed that the proportion of multi-drug resistant (MDR)-TB in smear-positive pulmonary TB patients was 8.32%. Of these, the proportion of MDR-TB in new patients and re-treated patients was 5.71% and 25.64%, respectively [4]. It is important to consider the incidence of drug-resistant TB in evaluating and improving disease prevention strategies in China [6].

The incidence of TB and MDR-TB varies widely across China because of the country’s vast geographic area, large population, uneven population distribution, varied climate types, and diverse levels of economic development [6–9]. The incidence of drug resistance is higher in the north, west, and central areas of China, and lower in south and east China [6, 9, 10]. Jilin Province is located in northeast China, with a total of 9 prefectures consisting of 60 counties. There are 27,494,100 individuals living in Jilin, of which, 45% belong to rural population. As one of the major agricultural province, Jinlin provides the largest per captia reserves of grain crop in China. In addition to agriculture, Jilin is also the national industry center for automobiles and train carriages. According to national policy, the suspected TB patients diagnosed in general hospital must be transferred to local TB control and prevention institution to receive the standardized treatment in Jilin Province. The free tuberculosis services are only provided for pulmonary tuberculosis patients seeking care in public health system, while this policy is not suitable for other medical institutions. Despite containment supports implemented by national authorities and the international community, the burden of TB and drug-resistant TB remains serious, while data on the incidence of drug-resistant TB in Jilin Province are not available at present. In 2008–09, the Jilin Province Center for Disease Control and Prevention (CDC) performed a survey of drug-resistant TB in Jilin Province to determine the proportion and incidence of drug-resistant TB and the risk factors associated with drug-resistant TB, especially MDR-TB. The goal was to fully understand the current status of and trends in TB incidence in Jilin Province and to provide a scientific basis for formulating and improving Jilin Province’s TB control and prevention program.

## Materials and Methods

### Ethics

The survey protocol was approved by the Ethical Committee of the Jilin Province CDC. All enrolled patients signed informed consent forms.

### Sampling Method

The TB Reference Laboratory of the Jilin Province CDC was responsible for implementing this survey. Following the cluster-randomized sampling method recommended by WHO guidelines for surveillance of drug-resistance in tuberculosis [11], the sample calculation for new smear-positive TB patients was based on the following hypothesis: the proportion of rifampin resistance was 9%, and the precision was ±1.8% when the confidence interval (CI) was 95%. For re-treated, smear-positive TB patients, the proportion of rifampin resistance was 16%, and the precision was ±3.2% when the CI was 95%. Accounting for possible loss due to culture failure and bacterial strains transport, the sample size was increased by 15%. It was estimated that the required sample size of new smear-positive TB patients was 1,165 cases, and that of re-treated, smear-positive TB patients was 605 cases.

Based on the required sample size and operability, the number of counties was confirmed as 30, and the distribution of sites was calculated according to the number of smear-positive TB patients newly registered in various cities at the prefecture level in 2007. Then the county TB control and prevention institutions of each prefecture were selected from corresponding prefecture as survey sites. The total population of the 30 counties (which include cities and districts) was 15,477,700 people, accounting for 56% of the province’s total population. On the basis of these calculations, every project site needed to enroll 40 new smear-positive TB patients and 21 re-treated, smear-positive TB patients.

People enrolled in the survey were new and re-treated, smear-positive pulmonary TB patients newly enrolled in local TB control and prevention institutions during the survey period. All the new cases or retreatment cases were consecutively enrolled, respectively. Because the enrollment of new cases was faster than that of retreated cases, the recruitment of new cases would be stopped if the new case number reached the requirement of sample size. A new pulmonary TB patient was defined as a patient who had never received anti-TB drug therapy or who had received therapy for less than one month. The re-treated pulmonary TB patient was defined as a patient who had received anti-TB drug therapy for more than one month.

### Patient Data Collection

All those administering the survey received training from the China CDC and the Jilin Province CDC. Each enrolled patient was independently interviewed by two survey administrators using the same questionnaire. The content of the questionnaire included basic societal data and treatment history (S1 Table). Patients with inconsistent interview results were reinterviewed and rechecked by a third survey administrator.

### Laboratory Examination

Each enrolled patient sent three sputum samples for smear microscopy and two sputum samples for mycobacterium culture. The cultures were done using the simple method on the acidic Lowenstein-Jensen (L-J) medium. An equal quantity of 4% NaOH was added into the sputum specimen, which was vortically shaken and digested for 15 minutes. Then 0.1ml of digested liquid was inoculated on the L-J medium and cultured in an incubator at 37°C. The culture tube was observed to exclude fast-growing microbes and other contaminants at three days and at one week after inoculation, and then observed weekly. Negative results were reported when no colony growth was observed at the end of the eighth week [4]. Positive cultures were sent to the Jilin Province TB Reference Laboratory to perform a conventional drug susceptibility test (DST) and bacterial species identification.

The DST was done by use of the proportion method on a solid L-J medium at the Jilin Province TB Reference Laboratory. The medium contained anti-TB drugs, and the final concentration of the drugs used was: 0.2 μg/ml for isoniazid, 40 μg/ml for rifampin, 4 μg/ml for streptomycin, 2 μg/ml for ethambutol, 40 μg/ml for kanamycin, and 2 μg/ml for ofloxacin. Mycobacterium species identification was done by growth test on a medium containing p-nitrobenzoic acid (PNB) and 2-thiophenecarboxylic acid hydrazide (TCH). The concentration of the drugs was: 500 mg/ml for PNB and 5 mg/ml for TCH [3, 4]. The Jilin Province TB Reference Laboratory has taken part in DST proficiency tests of the National TB Reference Laboratory since 2005, and the results of the test were qualified. Drug-resistant TB was defined as tuberculosis with drug resistance to any of the antituberculosis drugs in the survey. MDR-TB was defined as tuberculosis resistant to both isoniazid and rifampin, and extensive drug resistance (XDR)-TB was defined as tuberculosis resistant to at least isoniazid, rifampin, ofloxacin, and kanamycin [4].

### Statistical Analysis

All collected data were entered using Epi Data 3.02 software (EpiData Association, Odense, Denmark). In order to ensure accuracy, data were entered by two operators, and after entry, 10% of entered data were randomly selected and rechecked. The number of drug-resistant TB patients in Jilin Province was calculated using the method published by WHO [3, 12]. The incidence of drug-resistant TB in new TB patients was calculated by multiplying the estimated incidence of new tuberculosis cases of Jilin province in 2009 by the proportion of those cases that were drug-resistant. The incidence of re-treated drug-resistant tuberculosis was estimated by multiplying the estimated incidence of retreated tuberculosis cases of Jilin province in 2009 by the proportion of those cases that were drug-resistant [12]. Analysis was performed using SPSS14.0 software (SPSS Inc, Chicago, USA), and statistical description and statistical inference were conducted using the surveyfreq and surveylogistic process. The level of significance of univariate analysis was 0.05, and that for inclusion in the multivariate model was 0.15. The corrected R^2^ was used as the standard for model screening.

## Results

From October 1, 2008, to July 31, 2009, 1,830 TB patients were enrolled, including 1,200 new patients and 630 re-treated patients. Of these, 59 (3.2%) patients were not enrolled for final analysis due to negative cultures or contamination. The total of 1,771 TB patients included 1,174 new smear-positive TB patients and 597 re-treated, smear-positive TB patients. Together, these patients were more than the minimum sample size, which could represent the status of drug-resistant TB in Jilin Province.

### Incidence and Proportion of Drug-resistant TB Cases

In this survey, the proportion of new and re-treated, smear-positive TB patients resistant to at least one of the first-line anti-TB drugs was 32.2% and 53.0%, respectively. Overall, the proportion of new and re-treated, smear-positive TB patients resistant to four first-line anti-TB drugs (isoniazid, rifampin, ethambutol, and streptomycin) was 17.3% and 36.4% for isoniazid; 10.6% and 28.9% for rifampin; 4.4% and 11.9% for ethambutol; and 26.4% and 39.1% for streptomycin, respectively (Table 1).

**Table 1:** Resistance to first- and second-line antituberculosis drugs.

	New cases (N = 1174)		Re-treated cases (N = 597)	
	n (%)	95% CI	n (%)	95% CI
Susceptibility to all four first-line drugs^†^	796 (67.7)	65.1–70.4	280 (47.0)	43.0–51.0
Any resistance to first-line drugs	378 (32.3)	29.6–34.9	317 (53.0)	49.0–57.0
Isoniazid (INH)	202 (17.3)	15.1–19.4	218 (36.4)	32.5–40.3
Rifampin (RMP)	125 (10.6)	8.9–12.4	172 (28.9)	25.2–32.5
Ethambutol (EMB)	52 (4.4)	3.2–5.6	70 (11.9)	9.2–14.3
Streptomycin (SM)	310 (26.4)	23.9–28.9	233 (39.1)	35.2–43.0
Resistance to INH or RMP	126 (11.7)	9.8–13.7	113 (24.6)	20.7–28.6
Multidrug resistance (MDR)^‡^	101 (8.6)	7.0–10.2	138 (23.2)	19.8–26.5
Susceptibility to ofloxacin (OFX) and kanamycin (KM)	1084 (92.3)	90.7–93.8	505 (84.7)	81.8–87.6
Any resistance to OFX or KM	91 (7.7)	6.2–9.3	91 (15.3)	12.4–18.2
OFX resistance	66 (5.6)	4.3–6.9	68 (11.4)	2.7–4.8
KM resistance	44 (3.7)	2.7–4.8	43 (7.2)	5.1–9.3
MDR + resistance to OFX or KM	64 (5.6)	4.2–6.9	74 (12.8)	10.0–15.5
Extensive drug resistance^¥^	14 (1.2)	0.6–1.8	15 (2.5)	1.3–3.8

^†^ First-line antituberculosis drugs include isoniazid, rifampin, ethambutol, and streptomycin; second-line antituberculosis drugs include ofloxacin and kanamycin.

^‡^ Multidrug resistance was defined as resistance to at least isoniazid and rifampin.

^¥^ Extensive drug resistance was defined as resistance to at least isoniazid, rifampin, ofloxacin, and kanamycin.

With regard to the proportion of MDR-TB patients, 8.6% of new TB patients and 23.2% of re-treated TB patients were MDR-TB patients, resistant to both rifampin and isoniazid (Table 1). With regard to the proportion of XDR-TB patients, 13.9% of new MDR-TB patients and 10.9% of re-treated MDR-TB patients were XDR-TB patients. The proportion of new and re-treated MDR-TB patients resistant to one of the two second-line anti-TB drugs (ofloxacin and kanamycin) was 26.7% and 40.7%, respectively. Moreover, 1.2% of new smear-positive TB patients and 2.5% of re-treated, smear-positive TB patients were XDR-TB patients in Jilin Province (Table 1).

Consequently, we estimate that approximately 1,290 new MDR-TB cases (95% CI, 1070 to 1510) occur annually in Jilin Province, including 810 new TB patients (95% CI, 660 to 960) and 480 re-treated TB patients (95% CI, 410 to 550). It was also estimated that 160 XDR-TB cases (95% CI, 80 to 240) occur annually in Jilin Province, including 110 new TB patients (95% CI, 55 to 165) and 50 re-treated TB patients (95% CI, 25 to 75).

### Risk Factors for Drug-resistant TB

Based on patient treatment history, we analyzed the influence of various surveyed factors on drug resistance and multidrug resistance among new and re-treated TB patients. Among new TB patients, we found the various surveyed factors had no influence on the distribution of drug and multidrug resistance, including sex, age, ethnicity and contact history (*P*>0.05). For re-treated TB patients, several social and clinical characteristics, including sex, age and treatment history, also had no influence on the distribution of drug and multidrug resistance (*P*>0.05, S2 Table), while our statistical results showed that the risk factors for MDR-TB include being a retiree or unemployed, having an academic background above high school level, and having an annual income of more than 20,000 Yuan. The analysis also showed that the risk of MDR-TB for patients whose last treatment was performed in a general hospital was significantly lower than that of patients whose last treatment was performed in other medical institutions (*P* = 0.036) (Table 2). Multivariate analysis was performed on the above four factors, and the results showed that people with academic backgrounds above high school level and annual incomes of more than 20,000 Yuan were more susceptible to MDR-TB (Table 3).

**Table 2:** Univariate analysis of risk factors for drug-resistant tuberculosis (TB) in re-treated TB cases*.

Characteristics	A: Pan-sensitive TB^†^		B:Drug-resistant TB^‡^		Odds ratio [B/A] (95% CI)	P value [B/A]	C: Multidrug- resistant TB^¥^		Odds ratio [C/A] (95% CI)	P value [C/A]
	n/N	%	n/N	%			n/N	%		
Occupation										
Farmer	182/280	65.0	211/317	66.6	1.0 (Ref.)		79/138	57.2	1.0 (Ref.)	
Retired/unemployed	76/280	27.1	76/317	24.0	0.863 (0.593–1.255)	0.439	43/138	31.2	1.568 (1.019–2.412)	0.041
Worker and others^¶^	22/280	7.9	30/317	9.5	1.176 (0.655–2.111)	0.658	16/138	11.6	1.675 (0.835–3.361)	0.191
Education										
Illiteracy	30/280	10.7	31/317	9.8	1.0 (Ref.)		8/138	5.8	1.0 (Ref.)	
Elementary and middle schools	208/280	74.3	244/317	77.0	1.135 (0.665–1.938)	0.642	103/138	74.6	1.955 (0.901–4.245)	0.09
High school or above	42/280	15.0	42/317	13.2	0.945 (0.488–1.830)	0.866	27/138	19.6	3.194 (1.333–7.653)	<0.01
Annual income										
4200 yuan	111/273	40.7	129/304	42.4	1.0 (Ref.)		54/133	40.6	1.0 (Ref.)	
4200~20,000 yuan	148/273	54.2	149/304	49.0	0.860 (0.612–1.210)	0.388	63/133	47.4	0.931 (0.617–1.405)	0.735
>20,000 yuan	14/273	5.1	26/304	85.5	1.598 (0.796–3.210)	0.188	16/133	12.0	2.296 (1.139–4.631)	0.02
Medical institution providing last TB treatment										
TB dispensary system	131/280	46.8	155/317	48.9	1.0 (Ref.)		78/138	56.5	1.0 (Ref.)	
TB hospital	56/280	20.0	68/317	21.5	0.665 (0.441–1.002)	0.051	30/138	21.7	0.822 (0.507–1.330)	0.424
General hospital	72/280	25.7	84/317	26.5	0.981 (0.645–1.491)	0.927	21/138	15.2	0.564 (0.330–0.963)	0.036
Private hospital/clinic	6/280	2.1	5/317	1.6	0.915 (0.300–2.789)	0.876	2/138	1.4	0.513 (0.111–2.365)	0.392
Others	15/280	5.4	5/317	1.6	0.575 (0.256–1.294)	0.181	7/138	5.1	1.039 (0.421–2.567)	0.934
Supervisor for taking medicine										
Doctor	104/280	37.1	126/317	39.7	1.0 (Ref.)		62/138	44.9	1.0 (Ref.)	
Family	49/280	17.5	74/317	23.3	1.230 (0.788–1.920)	0.363	23/138	16.7	0.630 (0.367–1.079)	0.092
Patient	122/280	43.6	114/317	36.0	0.771 (0.536–1.110)	0.162	50/138	36.2	0.728 (0.475–1.116)	0.146
Others	5/280	1.8	3/317	0.9	0.495 (0.116–2.121)	0.344	3/138	2.2	1.626 (0.377–7.005)	0.514

* Only results for variables included in final multivariate model are presented; additional results are available in web-only supplement. Calculation of odds ratios takes into account the clustered design; therefore, the odds ratios presented in the table may not be reproducible simply from data in the table.

^†^ Pan-sensitive TB is tuberculosis that is susceptible to the four first-line antituberculosis drugs (isoniazid, rifampin, ethambutol, and streptomycin) in this survey.

^‡^ Drug-resistant TB is tuberculosis with drug resistance to any of the antituberculosis drugs in the survey.

^¥^ Multidrug-resistant TB is tuberculosis with resistance to both isoniazid and rifampin.

^¶^Others include students, detainers, teachers, doctors, businesspeople, and public officers.

**Table 3:** Multivariate analysis of risk factors for drug-resistant tuberculosis (TB) in re-treated TB cases.

	Drug-resistant TB*	Multidrug-resistant TB^†^
Characteristics	Adjusted odds ratio (95% CI)	Adjusted odds ratio (95% CI)
Retired/unemployed	1.382 (0.640–2.984)	1.333 (0.815–2.178)
High school or above	1.011 (0.482–2.119)	2.922 (1.200–7.114)
Annual income >20,000 yuan	1.624 (0.788–3.347)	2.107 (1.025–4.333)
TB hospital	1.071 (0.652–1.758)	0.613 (0.125–3.002)
General hospital	0.692 (0.445–1.077)	0.602 (0.322–1.125)
Family	1.309 (0.834–2.055)	0.739 (0.411–1.332)

*Drug-resistant TB is tuberculosis with drug resistance to any antituberculosis drugs in the survey.

^†^ Multidrug-resistant TB is tuberculosis with resistance to both isoniazid and rifampin.

### Analysis of Last Treatment Status of Re-treated TB Patients

**Fig 1:**
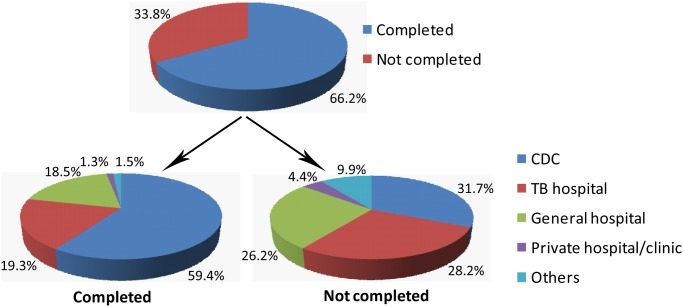
Proportion of previously treated tuberculosis patients who completed their last treatment course and the location of last treatment.

**Fig 2:**
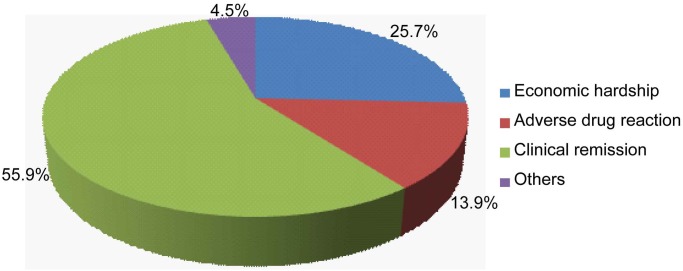
Reasons for treatment interruption among previously treated tuberculosis patients reported by the patients themselves. The clinical remission is patient rather than clincican defined.

Among 597 re-treated TB patients surveyed, 395 (66.2%) patients completed their final course of treatment, and 202 (33.8%) patients did not. Among patients who completed the last course of treatment, the proportions of medical institutions providing the last treatment were: 59.4% (TB control and prevention institutions of the CDC); 19.3% (TB hospitals); 18.5% (general hospitals); 1.3% (private clinics); and 1.5% (other medical institutions). Among patients who did not complete the last treatment course, the above proportions were 31.7%, 28.2%, 26.2%, 4.4%, and 9.9%, respectively (Fig. 1). Statistical analysis revealed that the distribution of re-treated patients between completed group and not completed group was significantly different (*P*<0.01). With regard to the 202 re-treated patients who did not complete the last treatment course, the first cause of discontinuing treatment was clinical remission deemed by the patient (55.9%), the second cause was economic hardship (25.7%), and the third cause was adverse drug reactions (13.9%) (Fig. 2).

## Discussion

The drug-resistance survey is an important measurement tool for understanding the proportion of drug resistance in a country or region and for formulating drug-resistant TB control strategies, and several regions with high TB burden have carried out the drug-resistance survey in recent years [2, 4, 13–15]. To our best knowledge, this report was the first study on the drug-resistance surveillance in Jilin Province. Data on the proportion of drug-resistant TB have also been reported in ten other provinces of China [16–18]. As compared with drug-resistance data in other provinces, the total drug-resistance status of Jilin Province among re-treated tuberculosis cases is comparable to the national level. But the proportion of rifampin resistance and MDR-TB in new tuberculosis cases is higher than at the national level and comparable to the level of surrounding regions (Liaoning Province, Heilongjiang Province, and Inner Mongolia Autonomous Region) (Tables 4 and 5). The drug resistance proportion among new and re-treated cases patients are considered as important indicators for TB epidemiology [8]. In Jilin, the higher number of MDR-TB among new cases suggests that the transmission of drug-resistant strains in Jilin is an urgent problem in the MDR-TB control and prevention system of Jilin Province. Unfortunately, basic laboratories in Jilin Province usually did not carry out cultures and conventional DSTs. The proportions of treatment failure, relapse, and secondary drug resistance increase when first-line drugs are used to treat drug-resistant patients. Early diagnosis of the above-mentioned patients is therefore essential to conduct effective individualized treatment. The use of the conventional DST in county-level laboratories is limited because of the additional time and biosafety requirements involved. Recently, WHO recommended the GeneXpert MTB/RIF test that can be used in the early detection of MDR-TB cases and HIV/TB cases (an MDR-TB–susceptible population) [17–20]. The application of molecular diagnosis tools may serve as a candidate solution for the dilemma between limit-resource setting and early diagnosis of drug-resistant tuberculosis in Jilin Province.

**Table 4:** Proportion of drug resistance in new tuberculosis cases tested in 11 Chinese provinces.

Province	Method of data collection	Number of patients tested	Any resistance	Resistance to rifampin	Multidrug resistance (MDR)^†^
North region in China					
Jilin	SVY*	1174	32.3%	10.6%	8.6%
Heilongjiang	SVY	1574	36.1%	10.6%	7.2%
Liaoning	SVY	818	42.1%	11.4%	10.3%
Inner Mongolia	SVY	806	35.0%	9.8%	7.3%
Beijing	SVY	1043	17.9%	4.2%	2.3%
Henan	SVY	646	35.0%	14.6%	10.9%
Shandong	SVY	1009	17.6%	3.8%	2.9%
South region in China					
Shanghai	SVY	764	15.4%	4.8%	3.9%
Zhejiang	SVY	809	14.8%	6.4%	4.4%
Guangdong	SVY	1432	18.0%	7.1%	5.4%
Hubei	SVY	859	17.5%	3.8%	2.0%
China^‡^	SVY	3037	34.2%	6.7%	5.7%

*SYV: drug resistance survey.

^†^ Multidrug-resistant TB is tuberculosis with resistance to both isoniazid and rifampin.

^‡^The data is cited from National Drug resistance Survey of China.

**Table 5:** Proportion of drug resistance in re-treated tuberculosis cases tested in 11 Chinese provinces.

Province	Number of patients tested	Any resistance	Resistance to rifampin	Multidrug resistance (MDR)^‡^
North region in China				
Jilin	597	53.0%	28.9%	23.2%
Heilongjiang	421	67.5%	40.4%	30.4%
Liaoning	86	55.8%	29.1%	24.5%
Inner Mongolia	308	70.1%	51.0%	41.9%
Beijing	154	35.1%	14.9%	11.7%
Henan	726	66.0%	43.5%	34.4%
Shandong	220	50.0%	25.1%	23.2%
South region in China				
Shanghai	200	27.5%	15.0%	12.5%
Zhejiang	145	59.3%	44.1%	34.5%
Guangdong	166	33.7%	19.9%	15.6%
Hubei	238	44.5%	26.9%	21.9%
China	892	54.5%	29.4%	25.6%

Previous studies have shown that among re-treated TB patients in China, risk factors for acquiring MDR-TB included being female and the number of treatments [21]. Our survey identified several unique risk factors for MDR-TB related to the regional features. First of all, the retirees and unemployed faced higher risks for MDR-TB in Jilin, which may be attributed to local climate and living habits. Jilin Province located in the cooler northeast region of China, where people tend to stay indoors for much of the year in buildings that may have poor ventilation in order to maintain a warmer indoor air temperature. In addition, the retirees were usually older than 60 years, and the weakened immune system of retirees may also responsible for high risk of MDR-TB. For the unemployed people, they always suffered great pressure and poor nutritional status, which served as another important reason for high risk. Furthermore, we found that people with higher academic backgrounds and higher incomes are at higher risk for MDR-TB. Numerous literature reports have shown that compliance with clinical treatment can be poor among people in these categories [22, 23]. The poor compliance and more prior treatments among the well-educated group possibly lead to MDR-TB.

Treatment discontinuation is considered as one of the risk factors for MDR-TB [24, 25–27]. In Jilin, patients whose treatment was not completed mainly came from TB hospitals and general hospitals. Although TB control and prevention institutions in the CDC system have professional follow-up systems, the patients from the hospital systems sometimes are missed due to the loss of transfer from hospital system to local TB dispensary system, who may not complete their follow-up treatment, serving as the high risk group of drug resistance [21]. Among patients whose last treatments were discontinued, the main causes were clinical remission as determined by the patient. This indicates that TB health education needs to be further strengthened. In addition, the economic hardship served as another major reason responsible for last treatment interruption. In Jilin, although the local TB dispensaries provide free treatment for tuberculosis patients, they must afford the cost for auxiliary treatment, including liver protectants and other drugs for reducing adverse reactions. The economic burden is higher for those receiving anti-TB treatments in hospital system, who have to afford more than 30% of the entire medical expenses in hospitals. Hence, economic assistance should be available to ensure TB patients with economic hardship can complete treatment, thus reducing the emergence of drug-resistant TB.

This survey has some limitations. First, this study enrolled the smear positive TB patients rather than all TB patients. Hence, the drug susceptibility profiles of smear negative/culture positive patients are still unknown. Second, patient HIV infection data were not collected. In China, routine HIV examination is not performed in TB patients, because the prevalence of HIV infection is assumed to be low. Due to higher HIV prevalence among TB patients than among the general population in China [28], HIV screening among TB patients will allow us to investigate the prevalence of HIV among tuberculosis patients in Jilin Province. Third, the patients seeking health care in the hospitals were not included in this study. As the high risk group of drug-resistant tuberculosis, the bias enrollment of those patients may result in the probable underestimation of the burden of drug-resistant tuberculosis in Jilin Province. Fourth, the burden of XDR tuberculosis was underestimated because only kanamycin rather than capreomycin or other second-line injectable aminoglycosides was used to identify XDR tuberculosis. Fifth, in this survey, all the risk factor data were obtained from patient interviews, which may have affected the accuracy of the data, a limitation that should be remedied in subsequent surveys. Sixth, the comorbidities in XDR-TB patient was another neglected issue in this study.

Overall, the survey results showed that Jilin Province remains one of the areas with the highest drug-resistant TB burden in China. The higher proportion of MDR-TB among new cases suggested that the transmission of drug-resistant strains in Jilin is an urgent problem in the MDR-TB control and prevention system of Jilin Province. Our results also revealed some risk factors for MDR-TB, including employment status, educational background, and income level. The survey also showed that health services should strengthen their systems to ensure better follow-up with TB patients to ensure they complete their course of treatment, and that strengthening health education and increasing financial investment could reduce the proportion of MDR-TB. The results provide a theoretical basis for formulating an appropriate Jilin Province TB control and prevention program.

## Supporting Information

S1 Table The questionnaire in this study (DOCX)

S2 Table Univariate analysis of risk factors (*P* value>0.15) for drug-resistant tuberculosis (TB) in re-treated TB cases (DOCX)
