# Supplementary material for: The Burden of MDR/XDR Tuberculosis in Coastal Plains Population of China
Source: PLoS One. 2015 Feb 17;10(2):e0117361. doi: 10.1371/journal.pone.0117361 (PMC4331508; doi:10.1371/journal.pone.0117361)
Supplement: S1 Table — (DOCX) [file pone.0117361.s001.docx]

**Table S1 The questionnaire in this study**

Pilot name： Pilot ID：

Patient name： Patient ID：

Address： Province County Town

Villiage Room

Registration ID：□□ □□□ Registered Date： Year Month Day

Classification：1 New case 2 Re-treated

**A Demographic information**

A1.Gender： 1　Male 2 Female

A2.Age： years

A3.Brith Date： Year Month Day

A4.Survey Date： Year Month Day

A5.Local living time： years

A6.Race：

1 Han 2 Mongolia 3 Hui 4 Tibetan 5 Uighur

6 Miao 7 Manchu 8 Koreans 9 Others

A7.Occupation：

1 Farmer 2 Worker 3 Bussiness 4 Medical

1. Education 6 Retiree 7 Unemployed 8 Student 9 Government 10 Others_____

A8.Education Level：

1 Illiteracy 2 Elementary 3 Middle

4 High 5 College

A9.Annual Income(RMB)：

1 <2500 2 2500~4200 3 4200 ~20000

4 20000 ~50000 5 >50000

A10.Number of family member：

**B Medical History**

B1. Symptoms when seeking health care this time.

1 Cough 2 Hemoptysis 3 Fever 4 Chest Pain 5 Others 7 None

B2. Have ever been diagnosed as tuberculosis (TB) patients?

1 Yes 2 No

***If the answer of B2 is “Yes”, please go to B3; If the answer of B2 is “No”, please go to C1.***

B3. What kinds of tuberculosis diseases? 1 Pulmonary 2 Extrapulmonary 3 Both

B4. The time of diagnosing as TB for the first time： Year Month

B5. Where were you diagnosed as TB for the first time?

1 General hospital 2 TB specialized hospital 3 TB dispensary/CDC 4 Private hospital 5 Individual clinic 6 Others

B6.Till now, have you ever received anti-TB treatment？

1 Yes 2 No

***If the answer of B6 is “Yes”, please go to B7; If the answer of B6 is “No”, please go to C1.***

B7.When did you receive the first anti-TB treatment: Year Month

B8.Till now, how many times have you ever received anti-TB treatment totally?

1 One 2 Two 3 Three and more

B9. How long have you received anti-TB treatment totally?

1 Less than one month 2 One month and more

B10. Where did you receive anti-TB treatment for the first time?

1 General hospital 2 TB specialized hospital 3 TB dispensary/CDC 4 Private hospital 5 Individual clinic 6 Others

B11.During the first anti-TB treatment period, who supervises you taking anti-TB drugs?

1 Doctor 2 Volunteer 3 Family 4 Oneself 5 Other

B12. Was there any adverse reaction during the first treatment period?

1 Yes 2 No

***If the answer of B12 is “Yes”, please go to B13; If the answer of B12 is “No”, please go to B14.***

B13. During the treatment period, when the adverse reaction emerged, how did you deal with it?

1 Continue the treatment after consulting the doctor

2 Stop the treatment after consulting the doctor

3 Stop the treatment without consulting the doctor

4 Continue the treatment without consulting the doctor

5 Others

B14.How long have you receive anti-TB treatment for the first time?

1 Less than six months 2 Six month 3 Eight months 4 More than eight months

B15. Has your treatment been interrupted before?

1 Yes 2 No

***If the answer of B15 is “Yes”, please go to B16; If the answer of B12 is “No”, please go to B20.***

B16. How many times has your treatment been interrupted before?

1 One 2 Two 3 Three or more

B17. Has your treatment been interrupted during the first anti-TB treatment?

1 Yes 2 No

B18. Why did your treatment been interrupted during the recent anti-TB treatment?

1 Economic hardship 2 I could not buy the anti-TB drugs

3 Adverse reaction 4 Clinical remission 5 Others

B19. Have your treatment been interrupted during the recent anti-TB treatment?

1 Yes 2 No

B20. Where did you receive health care during the recent anti-TB treatment?

1 TB dispensary/CDC 2 TB specialized hospital 3 General hospital 4 Private hospital 5 Individual clinic 6 Others

**C1 Classification**

1 New case 2 Re-treated case

Signature of doctor：

Date Year Month Day

Signature of the doctor for rechecking：

Date Year Month Day
