# Supplementary material for: The Burden of MDR/XDR Tuberculosis in Coastal Plains Population of China
Source: PLoS One. 2015 Feb 17;10(2):e0117361. doi: 10.1371/journal.pone.0117361 (PMC4331508; doi:10.1371/journal.pone.0117361)
Supplement: S2 Table — (DOCX) [file pone.0117361.s002.docx]

**Table S2. Univariate analysis of** **risk factors (P value>0.15) for drug-resistant tuberculosis (TB) in re-treated TB cases**

| Characteristics | A: Pan-sensitive TB^†^ | | B: Drug-resistant TB^‡^ | | Odds ratio  [B/A]  (95% CI) | P value [B/A] | C: Multidrug- resistant TB^¥^ | | Odds ratio  [C/A]  (95% CI) | P value [C/A] |
| --- | --- | --- | --- | --- | --- | --- | --- | --- | --- | --- |
|  | n/N | % | n/N | % |  |  | n/N | % |  |  |
| Sex |  |  |  |  |  |  |  |  |  |  |
| Male | 222/280 | 79.3 | 260/317 | 82.0 | 1.0 (Ref.) |  | 114/138 | 82.6 | 1.0 (Ref.) |  |
| Female | 58/280 | 20.7 | 57/317 | 18.0 | 0.839(0.559-1.261) | 0.407 | 24/138 | 17.4 | 0.806(0.476-1.364) | 0.513 |
| Age |  |  |  |  |  |  |  |  |  |  |
| <20 yr | 1/280 | 0.4 | 3/317 | 0.9 | 3.054(0.308-30.245) | 0.619 | 1/138 | 0.7 | 2.036(0.123-33.761) | 1.000 |
| 20-39 yr | 57/280 | 20.4 | 56/317 | 17.7 | 1.0 (Ref.) |  | 28/138 | 20.3 | 1.0 (Ref.) |  |
| 40-59 yr | 161/280 | 57.5 | 188/317 | 59.3 | 1.189(0.777-1.817) | 0.449 | 87/138 | 63.0 | 1.100(0.653-1.854) | 0.792 |
| ≥60 yr | 61/280 | 21.8 | 70/317 | 22.1 | 1.168(0.706-1.933) | 0.608 | 22/138 | 15.9 | 0.734(0.378-1.428) | 0.401 |
| Ethnicity |  |  |  |  |  |  |  |  |  |  |
| Han | 253/280 | 90.4 | 284/317 | 89.6 | 1.0 (Ref.) |  | 122/138 | 88.4 | 1.0 (Ref.) |  |
| Others | 27/280 | 9.6 | 33/317 | 10.4 | 1.089(0.637-1.861) | 0.786 | 16/138 | 11.6 | 1.229(0.638-2.366) | 0.608 |
| Contact History |  |  |  |  |  |  |  |  |  |  |
| No | 190/226 | 84.1 | 203/252 | 80.6 | 1.0 (Ref.) |  | 91/113 | 80.5 | 1.0 (Ref.) |  |
| Yes | 36/226 | 15.9 | 49/252 | 19.4 | 1.274(0.793-2.046) | 0.339 | 22/113 | 19.5 | 1.276(0.710-2.293) | 0.446 |
| Previous treatment times |  |  |  |  |  |  |  |  |  |  |
| 1 time | 221/274 | 80.7 | 245/306 | 80.1 | 1.0 (Ref.) |  | 99/122 | 81.1 | 1.0 (Ref.) |  |
| 2 times | 35/274 | 12.8 | 43/306 | 14.1 | 1.108(0.684-1.794) | 0.714 | 16/122 | 13.1 | 1.020(0.540-1.930) | 1.000 |
| ≥3 times | 18/274 | 6.6 | 18/306 | 5.9 | 0.902(0.458-1.777) | 0.863 | 7/122 | 5.7 | 0.868(0.351-2.145) | 0.826 |
| Times of interrupt treatment |  |  |  |  |  |  |  |  |  |  |
| 0 time | 179/280 | 63.9 | 205/317 | 64.7 | 1.0 (Ref.) |  | 91/138 | 65.9 | 1.0 (Ref.) |  |
| 1 time | 62/280 | 22.1 | 62/317 | 19.6 | 0.873(0.582-1.309) | 0.536 | 25/138 | 18.1 | 0.793(0.468-1.345) | 0.431 |
| 2 times | 22/280 | 7.9 | 26/317 | 8.2 | 1.032(0.565-1.884) | 1.000 | 12/138 | 8.7 | 1.073(0.508-2.265) | 0.849 |
| ≥3 times | 17/280 | 6.1 | 24/317 | 7.6 | 1.233(0.642-2.368) | 0.622 | 10/138 | 7.2 | 1.157(0.509-2.629) | 0.832 |
| Adverse reaction |  |  |  |  |  |  |  |  |  |  |
| No | 238/280 | 85.0 | 277/317 | 87.4 | 1.0 (Ref.) |  | 121/138 | 87.7 | 1.0 (Ref.) |  |
| Yes | 42/280 | 15.0 | 40/317 | 12.6 | 0.818(0.513-1.305) | 0.407 | 17/138 | 12.3 | 0.796（0.435-1.457） | 0.551 |

^†^ Pan-sensitive TB is tuberculosis that is susceptible to the four first-line antituberculosis drugs (isoniazid, rifampin, ethambutol, and streptomycin) in this survey.

^‡^ Drug-resistant TB is tuberculosis with drug resistance to any of the antituberculosis drugs in the survey.

^¥^ Multidrug-resistant TB is tuberculosis with resistance to both isoniazid and rifampin.
